# G6PD upregulates Cyclin E1 and MMP9 to promote clear cell renal cell carcinoma progression

**DOI:** 10.7150/ijms.58902

**Published:** 2022-01-01

**Authors:** Qiao Zhang, Yueli Ni, Shujie Wang, Yannick Luther Agbana, Qiaoqiao Han, Wenjing Liu, Honggang Bai, Zihan Yi, Xiaojia Yi, Yuzhi Zhu, Buqing Sai, Lijuan Yang, Qiong Shi, Yingmin Kuang, Zhe Yang, Yuechun Zhu

**Affiliations:** 1 Department of Biochemistry and Molecular Biology, School of Basic Medical Sciences, Kunming Medical University, Yunnan, Kunming 650500, P.R. China.; 2 Departments of Pathology, The First Affiliated Hospital of Kunming Medical University, Yunnan, Kunming 650032, P.R. China.; 3 Department of Clinical Laboratory, The Second Hospital of Jingzhou, Jingzhou, Hubei 434000, P.R. China.; 4 Department of Medical Oncology, The Third Affiliated Hospital of Kunming Medical University (Tumor Hospital of Yunnan Province), Yunnan, Kunming 650118, P.R. China.; 5 Department of Clinical Laboratory, The Third Affiliated Hospital of Kunming Medical University (Tumor Hospital of Yunnan Province), Yunnan, Kunming 650118, P.R. China.; 6 Departments of Organ Transplantation, The First Affiliated Hospital of Kunming Medical University, Yunnan, Kunming 650032, P.R. China.

**Keywords:** ccRCC, G6PD, Cyclin E1, MMP9, proliferation, migration

## Abstract

**Background:** Clear cell renal cell carcinoma (ccRCC) is a cell metabolic disease with high metastasis rate and poor prognosis. Our previous studies demonstrate that glucose-6-phosphate dehydrogenase (G6PD), the first and rate-limiting enzyme of the pentose phosphate pathway, is highly expressed in ccRCC and predicts poor outcomes of ccRCC patients. The aims of this study were to confirm the oncogenic role of G6PD in ccRCC and unravels novel mechanisms involving Cyclin E1 and MMP9 in G6PD-mediated ccRCC progression.

**Methods:** Real-time RT-PCR, Western blot and immunohistochemistry were used to determine the expression patterns of G6PD, Cyclin E1 and MMP9 in ccRCC. TCGA dataset mining was used to identify Cyclin E1 and MMP9 correlations with G6PD expression, relationships between clinicopathological characteristics of ccRCC and the genes of interest, as well as the prognosis of ccRCC patients. The role of G6PD in ccRCC progression and the regulatory effect of G6PD on Cyclin E1 and MMP9 expression were investigated by using a series of cytological function assays *in vitro*. To verify this mechanism *in vivo*, xenografted mice models were established.

**Results:** G6PD, Cyclin E1 and MMP9 were overexpressed and positively correlated in ccRCC, and they were associated with poor prognosis of ccRCC patients. Moreover, G6PD changed cell cycle dynamics, facilitated cells proliferation, promoted migration* in vitro*, and enhanced ccRCC development *in vivo*, more likely through enhancing Cyclin E1 and MMP9 expression.

**Conclusion:** These findings present G6PD, Cyclin E1 and MMP9, which contribute to ccRCC progression, as novel biomarkers and potential therapeutic targets for ccRCC treatment.

## Introduction

Clear cell renal cell carcinoma (ccRCC) is the most common and dangerous malignancy subtype derived from kidney tissue, accounting for approximately 80% of all renal cell carcinoma cases 1, 2. Globally, about 400,000 new diagnosed cases and 139,000 death cases are expected to occur per year 3. Accumulating evidences indicate that ccRCC is a cell metabolic disease with high metastasis rate, drug resistance and poor prognosis 4, 5. Over the last decades, although some patients with ccRCC can be diagnosed at early stages and cured by surgical resection, considerable number of ccRCC patients are still confronted with unfavorable prognosis because of high recurrence rate after surgical resection, and neither chemotherapy nor radiation therapy is effective for the patients with metastases 1, 6. Therefore, identifying key factors which are potentially recognized as diagnostic and prognostic biomarkers and functionally involved in ccRCC progression is still of great importance and may provide efficient diagnostic and therapeutic strategies for ccRCC patients.

Glucose-6-phosphate dehydrogenase (G6PD), the first and rate-limiting enzyme of the pentose phosphate pathway, is highly expressed in certain types of tumor, including lung cancer, breast carcinoma and RCC 7-9. It is the cornerstone of the metabolic reprogramming process in tumor cells that result in the increased production of building blocks necessary for nucleotides and lipids synthesis 10, 11. Previous studies from our research group demonstrate that G6PD overexpression is positively associated with ccRCC development and represents a potential prognostic factor for poor outcomes in ccRCC patients 9. Moreover, G6PD was found to promote ccRCC cell proliferation and invasion through upregulating the expression of Cyclin D1 and MMP2, respectively 9, 12. However, the molecular mechanisms underlying G6PD-mediated ccRCC development is not completely delineated.

Cell cycle regulatory factors are implicated in various stages of tumorigenesis 13, and aberrant expression of the molecules that regulate the G1/S phases transition has been observed in different types of malignancies, including RCC 14, implying that cell cycle defects are linked to the activation of oncogenes. However, the presence and underlying mechanisms of aberrate G1/S regulatory molecules have only partly been clarified in ccRCC. Cyclin D1 and Cyclin E1 are two crucial G1/S transition regulatory factors that are often deregulated and play oncogenic roles in tumor proliferation and progression 14. It has been reported that Cyclin D1 is abnormally highly expressed in ccRCC and promotes cell proliferation by regulating cell G1/S transition 14, 15. Similarly, the protein expression of Cyclin E1 has also been reported to be higher in RCC and associated with RCC tumor behavior. High Cyclin E1 level is positively correlated with RCC aneuploidy, staging and nuclear grade. There is also an association between Cyclin E1 and the S-phase fraction and high levels of Cyclin E1 is positively associated with rapid RCC proliferation 14.

Metastasis is a complex process that involve the participation of different key genes. Extracellular matrix (ECM) remodeling is crucial for the cell adherence at the initiation of the tumor metastatic stage and matrix metalloproteinases (MMPs) are strongly implicated in the degradation of the ECM, emphasizing their crucial roles in tumor metastasis 16. MMPs expression and activity are upregulated in certain carcinomas where they exert important roles in cancer metastasis. Especially, MMP2 and MMP9, members of the MMPs, have been found to be significantly overexpressed in RCC and involved in RCC metastasis and angiogenesis 17-19.

To the best of our knowledge, the molecular mechanism of G6PD regulating Cyclin E1 and MMP9 involvement in ccRCC progression has not yet been untangled. Here, we uncover the clinicopathological implications of G6PD, Cyclin E1 and MMP9 in ccRCC. Hence, functional and mechanistic analyses help to unravel a novel mechanism of G6PD-mediated ccRCC progression. In addition, Cyclin E1 and MMP9 show more potential implication in ccRCC progression than Cyclin D1 and MMP2 respectively.

## Materials and Methods

### Human specimens and immunological histological chemistry (IHC) analysis

A total of 20 pairs of ccRCC tumor specimens and matched adjacent normal tissues were obtained from ccRCC patients without any treatment before surgery at the Department of Organ Transplantation of the First Affiliated Hospital of Kunming Medical University. The obtained specimens were sectioned, embedded in paraffin at the Department of Pathology of the First Affiliated Hospital of Kunming Medical University and then used for IHC analysis. Informed consent was obtained from the patients and the study was approved by the Ethics Committee of Kunming Medical University, according to the regulations of the Declaration of Helsinki.

For IHC analysis, the paraffin-embedded tissue sections were firstly dewaxed. Next, endogenous peroxidase was removed by 3% H_2_O_2_ for 10 min at room temperature. IHC was conducted by using General-purpose two-step detection kit (PV-9000, ZSGB-BIO, Beijing, China) according to the manufacturer's protocol. The following antibodies were used: G6PD antibody (ab133525, Abcam), Cyclin E1 antibody (bsm-52048R, Bioss, Beijing, China), MMP9 antibody (ab76003, Abcam). At last, tissues were stained by using DAB detection kit (Amplifier polymer) (DAB-2031, MXB Biotechnology, Fuzhou, China) for an appropriate time, dehydrated, mounted and photographed. The staining score which ranged from 0-12 points was calculated by the staining intensity multiplied by the percentage of stained cells as described before 9, 12. Staining intensity could be classified into negative (0 point), weak (1 point), moderate (2 points) and strong (3 points). Percentage of positive stained cells was designated into four types: 1 point as < 25%, 2 points as 26~50%, 3 points as 51~75% and 4 points as > 75%. Final staining scores over 4 points were considered high expression.

### TCGA data analysis

Using R software to login The Cancer Genome Atlas database. The transcriptome data from 535 clear cell renal cell carcinoma cases and 72 normal kidney tissues (Kidney renal clear cell carcinoma [KIRC]) were harvested and the clinicopathological information of patients were matched, of which only 528 patients had expression profile data and prognostic survival information, including survival time and survival status. We used KIRC gene expression profile data (Fragments Per Kilobase of transcript per Million fragments mapped, FPKM) for gene expression differences analysis. The downloaded Counts data was converted by log_2_ (FPKM+1). Quasi-Likelihood F-tests method of the package of EdgeR software 20 was used to analyze the expression difference of Cyclin D1, Cylin E1 and MMP9 between ccRCC and normal control tissues.

### Cell culture and stable cell construction

The most highly cited and commonly used cell lines for ccRCC researches, including 786-O, ACHN and Caki-1 cells 2 were bought from Kunming institute of zoology, Chinese academy of sciences. Cell thawing was firstly conducted at 37℃ water bath, centrifuged, removed cell freezing medium in ultra clean bench and transferred cells to the culture bottle. DMEM culture medium (1195500 bt, GIBCO, USA) containing 10% FBS (10099141, GIBCO, USA) was added. Culture conditions were 37 ℃, 5% CO_2_ and saturated humidity. When cell convergence was about 80%, the culture medium was abandoned, cells were washed with PBS for twice, and 1 mL of 0.25% of trypsin (25200072, GIBCO, USA) was added to digest cells for 1 ~ 2 min. When most of the cell fell off, fresh culture medium containing serum was added and transferred to new culture bottles by 1:3.

Our previous results showed that Caki-1 cells had the highest G6PD activities in the three of RCC cell lines, which were much higher than that of the control HK2 cell lines. Whereas, 786-O and ACHN had the moderate and lowest activities of G6PD, respectively. Therefore, G6PD-overexpressing ACHN and 786-O or G6PD-knocked down Caki-1 and 786-O stable cells establishment were conducted as described before 9, 12, 21. For G6PD-overexpressing ACHN cells construction, 2 x 10^5^ ACHN cells were firstly seeded in a 6-well culture plate. When reaching 70-80% confluence, cells were transfected with 2 μg pBABE-puro-G6PD or the control plasmid using Lipofectamine 2000. 48h after transfection, 0.5 μg/ml puromycin was used for 3 weeks resistance selection. To construct G6PD-knocked down Caki-1 stable cells, 2 x 10^5^ Caki-1 parental cells were firstly seeded in a 6-well culture plate. When reaching 70-80% confluence, cells were transfected with 2 μg pSR-GFP/Neo-G6PD shRNA or the control plasmid using Lipofectamine 2000. 48h after transfection, 1000 μg/ml G418 was used for 3 weeks resistance selection.

### Real-time RT-PCR and Western blot analysis

For real-time RT-PCR assay, total RNA was extracted from cells or tissues according to Trizol (15596-018, Invitrogen, USA) reagent instructions. cDNA was synthesized by reverse transcription according to Thermo RT Kit (K1622, Thermo, USA) instructions. Real-time PCR was performed using SYBR Green qPCR SuperMix (04913850001, Roche, Switzerland). Primers used were as follows: G6PD: F: 5'-TCATCATCATGGGTGCATCGG-3', R: 5'-CTTGAAGAAGGGCTCACTCTGTTTG-3'; Cyclin D1: F: 5'-GCGTACCCTGACACCCCTCTC-3', R: 5'-CTCCTCTTCGCCTGATCC-3'; Cyclin E1: F: 5'- ACTCAACGTGCAAGCCTCG-3', R: 5'- GCTCAAGAAAGTGCTGATCCC-3'; MMP9: F: 5'- AATCTCTTCTAGAGACTGGGAAGGAG-3', R: 5'-AGCTGATTGACTAAAGTAGCTGGA-3'; U6: F: 5'-CTCGCTTCGGCAGCACA-3', R: 5'-AACGCTTCACGAATTTGCGT-3'.

For Western blot analysis, the total protein of cells or tissues was extracted with radioimmunoprecipitation assay (RIPA) protein lysis buffer (supplemented with PMSF) and quantified by bicinchoninic acid (BCA) method. SDS-PAGE with 10% separation gel and 5% compression gel were prepared. Equal amount of protein was loaded, and electrophoresis, membrane transfer and blocking with 5% non-fatty milk were performed. Afterward, the membranes were incubated with primary antibodies and then secondary antibody. The following antibodies were used: G6PD antibody (ab133525, Abcam, Cambridge, U.K.), Cyclin D1 antibody (ab16663, Abcam), Cyclin E1 antibody (ab33911, Abcam), MMP9 antibody (ab76003, Abcam), β-actin (#4967, Cell Signaling Technology, Beverly, MA, USA), goat anti‑rabbit IgG secondary antibody (sc2004, Santa Cruz Biotechnology) and goat anti‑mouse IgG secondary antibody (sc2005, Santa Cruz Biotechnology). The results were detected by chemiluminescence method after washing the film with TBST solution. The results were analyzed by Image J grayscale scanning software.

### Cell cycle and proliferation assay

For cell proliferation detection, 100 μL cells suspension (1×10^4^/well) were seeded into 96-well plates for 24 h. 20 μl of MTS reagent (CTB169, Promega, Beijing, China) was added to each well at different time points, and incubated for 2 h at 37 ℃, followed by the measurement of absorbance at 490 nm using a microplate reader.

For cell cycle assay, cells were firstly seeded into 6-well plates and grown for 12 h. After cultured in 0.2% FBS medium for 24 h, cells were incubated in 10% FBS medium for another 24 h. Cells were harvested and cell cycle analysis were performed as described in a previous report by a PARTEC CyFlow Space flow cytometer and ModFit software 12.

siRNA-Ctrl and siRNA-targeting Cyclin E1 (sequences: 5'-CACCCTCTTCTGCAGCCAA-3') were synthesized by Ribobio (Guangzhou, China). For siRNA transfection, 2 x 10^5^ ACHN cells were firstly seeded in a 6-well culture plate. When reaching 70-80% confluence, cells were transfected with 10 nM siRNA-Ctrl or siRNA-targeting Cyclin E1 using Lipofectamine 2000. 48 h after transfection, cells were harvest and used for the following cell cycle, MTS and other experiments.

### Wound healing and Transwell assay

For wound healing analysis, 1×10^5^ cells were seeded into 6-well plates and cultured until 90% confluency. A 200 μL pipette tip was used to make three parallel wounds in each well, and all wells were washed by PBS for twice. Cells were then cultured in serum-free medium and images were captured by inverted microscopy at 0 and 24 h after scratching. Cell migration distances were analyzed by using ImageJ software.

For Transwell migration analysis, 1×10^5^ cells in 100 μl of serum-free medium were seeded on the top surface of a 24-well (8 μM Transwell membranes) and 600 μl medium with 10% FBS was added in the bottom of Transwell chambers. After incubation for 24 h, the Transwell membranes were fixed with 4% formaldehyde for 20 min and stained with Crystal Violet for 10 min at room temperature. At last, cell numbers of 10 areas of each Transwell membrane, observed at 400× magnification, were analyzed and cell migration abilities of each cell line were assessed.

MMP9 specific inhibitor JNJ0966 was purchased from Selleck (S5696, Houston, TX, USA) and dissolved in 100% DMSO to prepare a 10 mM stock and stored at -20°C. For cell treatment, the stock solution was added in the culture medium at a final concentration of 10 μM. Following 24 h stimulation, the migratory abilities of ccRCC cells were analyzed by Transwell assay as described above.

### Mice model

A total of 20 six-week old BALB/c nude mice were purchased from the Beijing HFK Bioscience Co., Ltd (Beijing, China) and housed under pathogen-free conditions. All animal experiments were performed according to the guidelines of Animal Care and Use Committee of Kunming Medical University. A total of 20 mice were randomly divided into 4 groups and they were subcutaneously injected into their flanks with 1 × 10^6^ ACHN- G6PDOE, Caki-1- G6PDsi, or relevant control cells, respectively. Tumor sizes were monitored every 5 days by using formula: (length × width^2^) × 0.5. The mice were euthanized after the last measurement and tumors were collected for further studies.

### MMP9 activity assay

For MMP9 activity assay in stable transfected cells and xenograft mice model tissues, MMP9 assay kit for cell (GMS50088.1, Genmed, Shanghai, China) and MMP9 assay kit for tissue (GMS50088.2, Genmed, Shanghai, China) were used, respectively. This assay was dependent on the following principles: as matrix metalloproteinase substrate, gelatin was chemically modified by succinic anhydride to block its free amino group to react with 2,4,6-trinitrobenzenesulfonic acid; Once hydrolyzed by MMP9, major amines, including new reactive amino groups, are released and exposed and react with trinitrobenzene sulfonic acid to produce a chromatic reaction. Therefore, MMP9 activity was measured following the manufacturer′s instruction and absorbances at OD 420 nm were detected by using spectrophotometer at 37˚C. The specific activity of MMP9 was calculated according to the gross activity subtract the nonspecific activity.

### Statistical analysis

SPSS version 21.0 (IBM, Armonk, NY) was used for data statistical analysis. As the TCGA data was not normal distribution and the variance was uneven, the correlation between the expression of Cyclin D1, Cyclin E1, MMP9 and clinical parameters of ccRCC patients was analyzed by Mann-Whitney U test (two groups) or Kruskal-Wallis H (K) test (three groups). The survival analysis was investigated by Kaplan-Meier curves, and log-rank test was performed to measure the statistical difference. Genes high and low expression groups were made by employing the median cutoff values. Univariate and multivariate Cox regression models of survival were applied to analyze the prognostic values of genes expression and clinicopathologic features. The *χ*^2^ test was used for IHC analyses. Spearman correlation analysis was conducted by using TCGA data of both the normal and tumor tissues to evaluate the expression correlation between two different molecules. For other analysis, unpaired or paired Student's t‑test was used. Error bars represent the means ± standard deviation. *p* < 0.05 indicates a significant statistical difference.

## Results

### G6PD, Cyclin E1 and MMP9 are overexpressed in ccRCC and associated with poor outcomes in ccRCC patients

**Figure 1:**
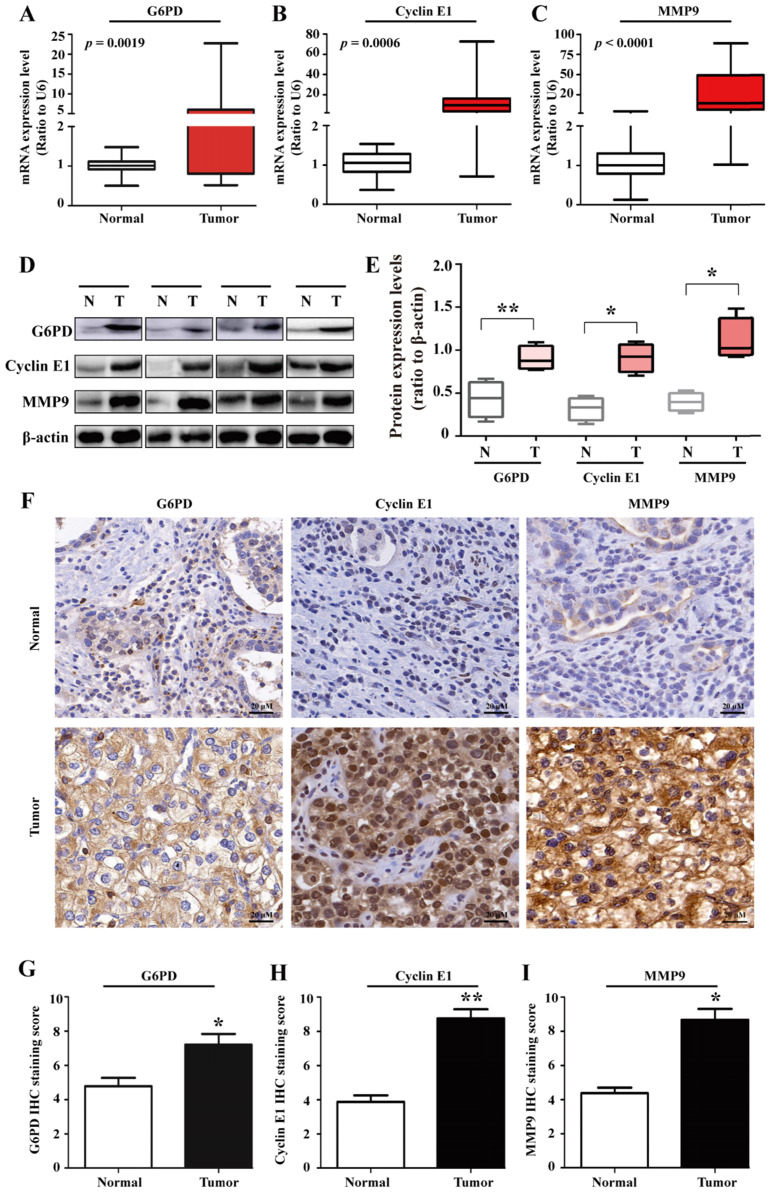
** G6PD, Cyclin E1 and MMP9 are overexpressed in human ccRCC tissues.** (**A-E**) real-time RT-PCR (**A-C**), Western blot (**D**) and grayscale scanning (**E**) were employed for the detection of G6PD, Cyclin E1 and MMP9 expression levels in ccRCC tumor specimens and relevant adjacent normal tissues (n=20). β-actin was used as a loading control. Representative cropped gels and blots of the Western blot analysis were shown (**D**). The samples used for quantitative comparisons in the Western blot analysis were derived from the same experiment and that gels were processed in parallel (**E**). (**F-I**) IHC were conducted to analyze the expression of G6PD, Cyclin E1 and MMP9 in ccRCC and relevant adjacent normal tissues (n=20). Representative images were shown (**F**). Statistical analysis was conducted by paired Student's *t*-test for Western blot analysis (**E**) and by *χ*^2^ test for IHC analysis (**G-I**), respectively. *p <0.05, **p <0.01 vs. Normal tissues.

To further unravel the underlying mechanisms of G6PD in ccRCC progression, 20 pairs of ccRCC tumor specimens and matched adjacent normal tissues were assessed by real-time RT-PCR, Western blot and IHC analysis. The results showed that the expression of G6PD, Cyclin E1 and MMP9 at both mRNA and protein expressions levels were elevated in human ccRCC tumors compared with adjacent normal tissues **(Fig. 1A-I)**, indicating that highly expressed Cyclin E1 and MMP9 may be positively correlated with G6PD overexpression and synergistically involved in ccRCC tumorigenesis.

**Figure 2:**
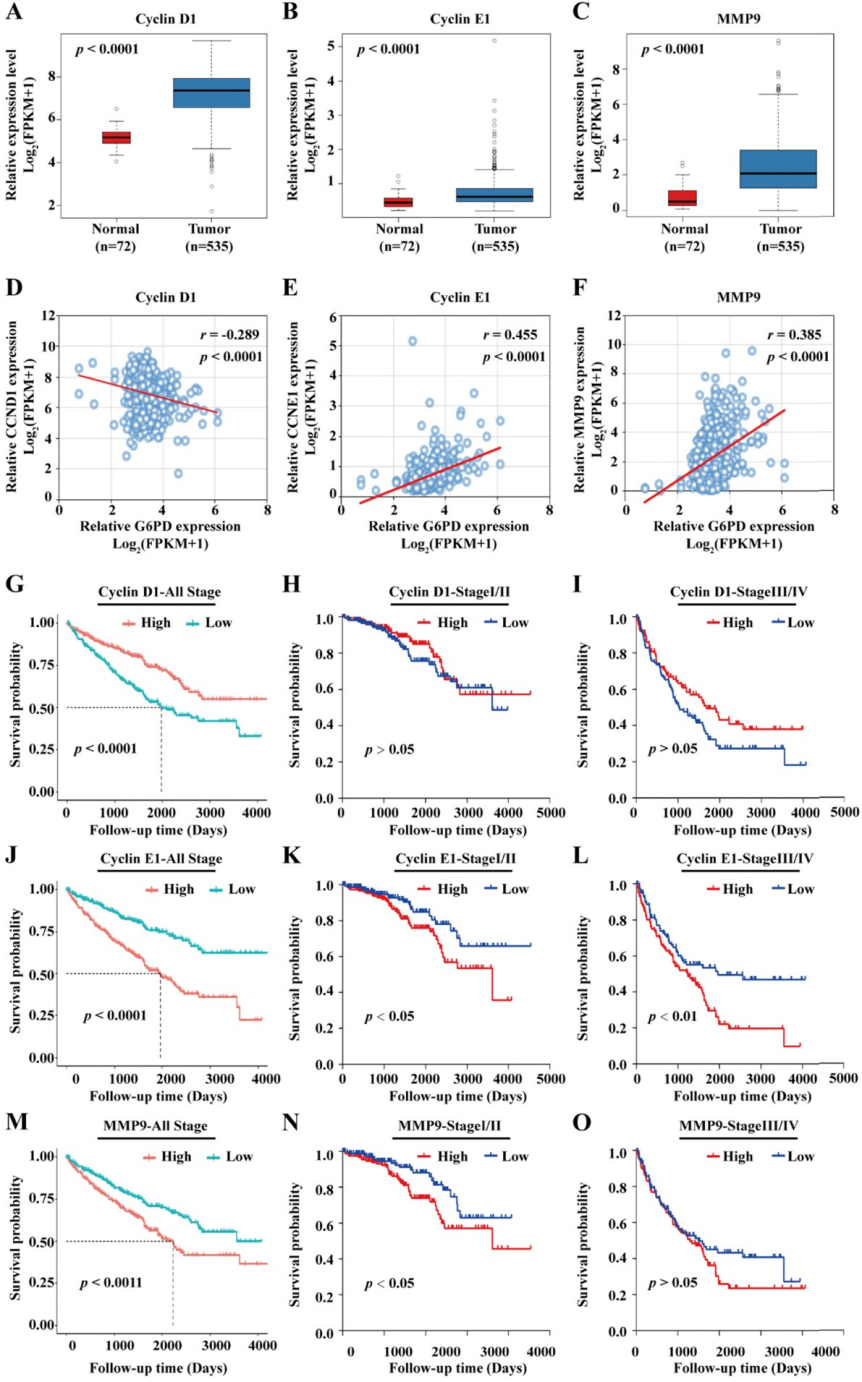
** Cyclin E1 and MMP9 are positively correlated with G6PD and associated with poor outcomes in ccRCC patients.** (**A-C**) mRNA expression levels of Cyclin D1, Cyclin E1 and MMP9 in normal kidney tissues (n=72) and ccRCC specimens (n=535) were analyzed by TCGA dataset mining (Mann-Whitney U test). (**D**-**F**) Spearman correlation analyses between G6PD and Cyclin D1, G6PD and Cyclin E1, G6PD and MMP9 at the mRNA expression levels were conducted in ccRCC and normal kidney tissues. (**G-O**) Kaplan-Meier analyses for overall survival of all ccRCC patients (n=528), patients with stage I/II ccRCC (n=320) and patients with stage III/IV ccRCC (n=205) in the TCGA cohort with high vs. low indicated gene mRNA expression levels were shown (log-rank test).

Previous studies from our research group demonstrate that G6PD could promote ccRCC cell proliferation and invasion through upregulating the expression of CyclinD1 and MMP2, respectively 9, 12. Therefore, transcriptome sequencing data of 72 normal kidney tissues and 535 ccRCC cases were subsequently extracted from TCGA and subject to statistical analyses for further evaluating the expression profile and the role of the genes of interest including Cyclin D1, Cyclin E1, MMP2 and MMP9. The results of gene expression analyses showed that Cyclin D1, Cyclin E1 and MMP9 mRNA levels were significantly higher in ccRCC than that in normal tissues **(Fig. 2A-C)**, whereas there was no significant difference between the expression level of MMP2 in ccRCC and normal control tissues** (Supplement 1A)**. Moreover, MMP2 expression was not associated with ccRCC prognosis** (Supplement 1B)**, indicating that G6PD mediated ccRCC progression may be depended on other more important underlying mechanisms. Subsequently, correlation analysis between the expression level of Cyclin D1, Cyclin E1, and MMP9 and clinicopathological features was performed. We observed a significant association between the expression levels of the three genes and the pathologic T stage, Fuhrman grade and TNM stage. However, only the expression levels of the proliferation-related genes Cyclin D1 and Cyclin E1 were significantly associated with the lymph node metastasis, and only the Cyclin E1 and MMP9 expression levels showed significant correlation with distant metastasis (M stage). Additionally, the expression levels of the three genes were significantly associated with the expression of G6PD in ccRCC specimens, indicating that all these genes may interact with G6PD in ccRCC tumorigenesis **(Table 1)**.

To further examine the association between G6PD and these three genes, spearman correlation analysis was conducted using the TCGA data. As presented in **Fig. 2D-F**, the results showed that G6PD is positively correlated with Cyclin E1 (*r* = 0.455; *p* < 0.001) and MMP9 (*r* = 0.385; *p* < 0.001), but rather negatively correlated with Cyclin D1 (*r* = -0.289; *p* < 0.001); suggesting that Cyclin E1 and MMP9 overexpression may be dependent on G6PD dysregulation in ccRCC. Taken together, these results indicate that these proliferation-and metastasis-related factors, especially Cyclin E1 and MMP9, might be involved in G6PD mediated ccRCC progression, and correlated with ccRCC prognosis.

To evaluate the prognostic significance of the genes in ccRCC, all the 528 ccRCC cases obtained from the TCGA were divided into high and low expression groups based on the median value of genes expression levels, Kaplan-Meier overall survival curves were plotted and log-rank test were conducted. The results demonstrated that patients with high Cyclin D1 expression level displayed a better prognosis **(Fig. 2G)**. In addition, when the patients were separated into stage I/II (n=320) and stage III/IV (n=205) according to the TNM staging (3 patients with no specific staging in all the 528 cases), no significant association between Cyclin D1 expression and patients' survival was observed **(Fig. 2H-I)**. Conversely, ccRCC patients with higher expression levels of Cyclin E1 and MMP9 had significantly shorter survival time than patients with low Cyclin E1 and MMP9 expression levels **(Fig. 2J, M)**. Similarly, higher Cyclin E1 and MMP9 expression levels predicted worse survival rate in both ccRCC stage I/II and stage III/IV **(Fig. 2K-L, N-O)**.

Furthermore, univariate Cox regression analysis revealed that high expression levels of G6PD, Cyclin E1 and MMP9, age at surgery, pathologic T stage, M stage, Fuhrman tumor grade, tumor laterality, as well as TNM stage were significant predictors of poor overall survival in ccRCC patients, whereas gender and N stage failed to be prognostic factors **(Table 2)**. In addition, multivariate Cox regression analysis demonstrated that the expression of G6PD and Cyclin E1, as well as age at surgery, M stage and TNM stage were independent prognostic factors for ccRCC overall survival **(Table 2)**. Taken together, these results indicate that G6PD, Cyclin E1 and MMP9 might play crucial role in the progression of ccRCC.

### G6PD upregulates the expression of Cyclin E1 and MMP9 *in vitro*

**Figure 3:**
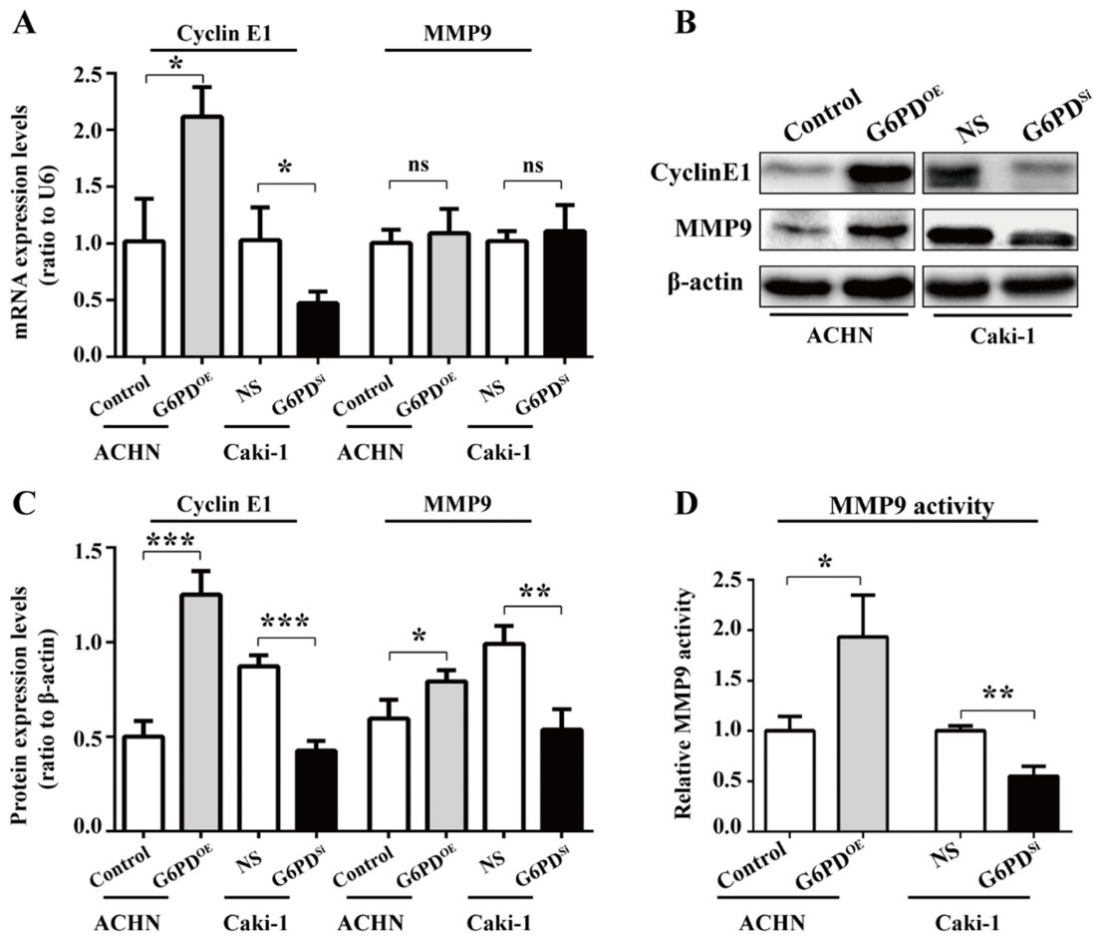
** G6PD upregulates the expression of Cyclin E1 and MMP9 *in vitro*.** (**A-C**) The expression of Cyclin E1 and MMP9 at the mRNA and protein level in stably transfected ACHN-G6PD^OE^, Caki-1- G6PD^si^ and relevant control cells was analyzed by using real-time RT-PCR (**A**), Western blot and grayscale scanning assay (**B-C**), respectively. β-actin was used as a loading control. Representative cropped gels and blots of the Western blot analysis were shown (**B**). The samples used for quantitative comparisons in the Western blot analysis were derived from the same experiment and that gels were processed in parallel (**C**). (**D**) Relative MMP9 enzyme activities in ACHN-G6PD^OE^, Caki-1- G6PD^si^ and relevant control cells were analyzed by using MMP9 activity kit in stable transfected ACHN or Caki-1 cells. All assays were done in at least triplicate. Bars represent the means ± SD. *p <0.05, **p <0.01, ***p <0.001 vs. Control or Non-silencer (unpaired Student's *t*-test).

To confirm that the interplay between G6PD and aforementioned genes is necessary for ccRCC progression, we first evaluate the related genes expressions in ACHN-G6PD^OE^, Caki-1-G6PD^si^ and control cells by real-time RT-PCR and Western blot respectively. The results demonstrated that G1/S transition- and proliferation-related gene Cyclin E1 was significantly increased by approximately 1.1-fold at the mRNA level plus 1.5-fold at the protein level in ACHN-G6PD^OE^ cells, whereas the expression levels of Cyclin E1was reduced by about 54.1% at the mRNA level plus 51.2%, respectively at the protein level in Caki-1-G6PD^si^ **(Fig. 3A-C)**.

Regarding the cell migration-related gene, our results showed that MMP9, the matrix metalloproteinase which exhibited the largest fold change between ccRCC and normal control tissues 17, had not been changed significantly at the mRNA level when G6PD was overexpressed or knocked down. In contrast, the Western blot results showed that MMP9 was significantly increased by about 2-fold in ACHN-G6PD^OE^, whereas it was decreased by 45.6% in Caki-1-G6PD^si^ **(Fig. 3A-C)**. Furthermore, the enzyme activity analysis also demonstrated that when G6PD was overexpressed, a 0.9-fold increase of MMP9 activity was detected in ACHN-G6PD^OE^ cells compared with the control, whereas G6PD-knockdown resulted in an approximately 45.0% of MMP9 activity reduction in Caki-1-G6PD^si^ cells compared with control cells **(Fig. 3D)**. Overall, these results suggest that G6PD-mediated ccRCC progression probably require the upregulation of Cyclin E1 and MMP9.

### G6PD changes cell cycle dynamics and facilitates ccRCC cells growth

**Figure 4:**
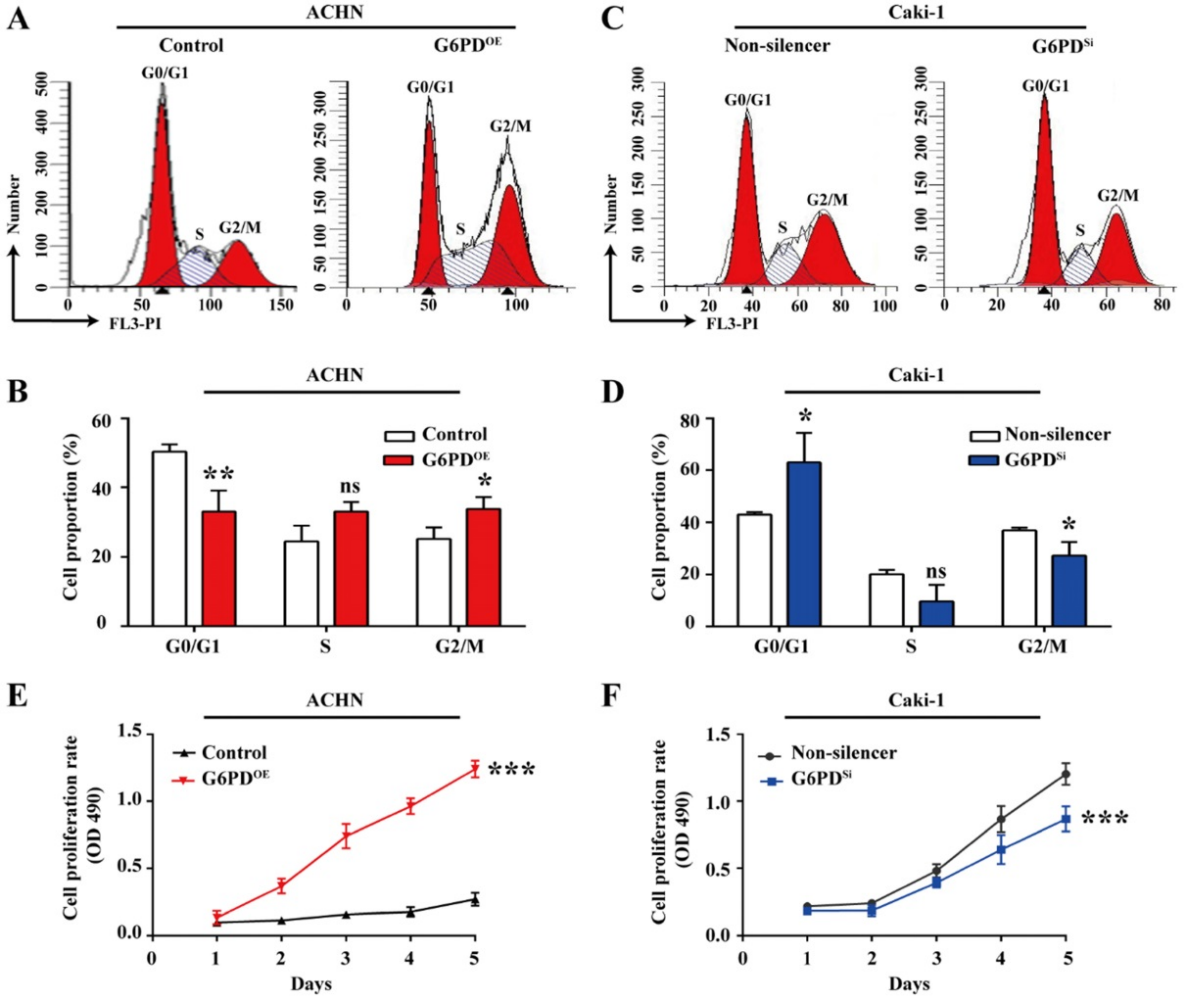
** G6PD changes cell cycle dynamics and facilitates ccRCC cells growth.** (**A-D**) Stably transfected ACHN-G6PD^OE^, Caki-1- G6PD^si^ and relevant control cells were subjected to cell cycle distribution analysis by PI staining and flow cytometry assay. (**E-F**) Cell proliferation abilities of ACHN-G6PD^OE^, Caki-1- G6PD^si^ and relevant control cell lines were assessed by MTS assay at different time points (1~5 day). The statistical data represented three independent experiments, each performed in triplicate. Error bars represent the means ± SD. ns, Not significant, *p <0.05, **p <0.01, ***p <0.001 vs. Control or Non-silencer group (unpaired Student's *t*-test for **B-C**, Mixed ANOVA for **E-F**).

Given that G6PD and Cyclin E1 are overexpressed and positively correlated, we aimed to elucidate their possible interplay in ccRCC cells proliferation. To do so, the cell cycle profiles were analyzed in ACHN- G6PD^OE^, Caki-1- G6PD^si^ and relevant control cells. As presented in** Fig. 4A-B**, in ACHN- G6PD^OE^ cells, the cell population of G0/G1 phase was significantly decreased by approximate 34.3%, while the cell population of S and G2/M phases showed an obvious increase compared to that of the control. In contrast, G6PD-knockdown (Caki-1- G6PD^si^) resulted in a 0.3-fold increase in the G0/G1 fraction and a decrease in the S and G2/M phase compared to that of the control (Non-silencer) **(Fig. 4C-D)**. These results indicated that G6PD might promote ccRCC cells proliferation through promoting the G1/S transition and changing the cell cycle distribution. The results of subsequent MTS assay confirmed that overexpression of G6PD in ACHN cells significantly increased the cell growth rate by about 3.6-fold at day 5 after seeding compared to that of the control cells **(Fig. 4E)**. Meanwhile, when G6PD was knocked down, an approximate 27.8% decreased proliferation rate was observed in Caki-1-G6PD^Si^ cells at day 5 compared to that of the Non-silencer cells **(Fig. 4F)**. Taken together, these results suggest that G6PD might facilitate ccRCC cells proliferation through the regulation of cell cycle progression by modulating Cyclin E1 expression.

### G6PD enhances the migration ability of ccRCC cells

**Figure 5:**
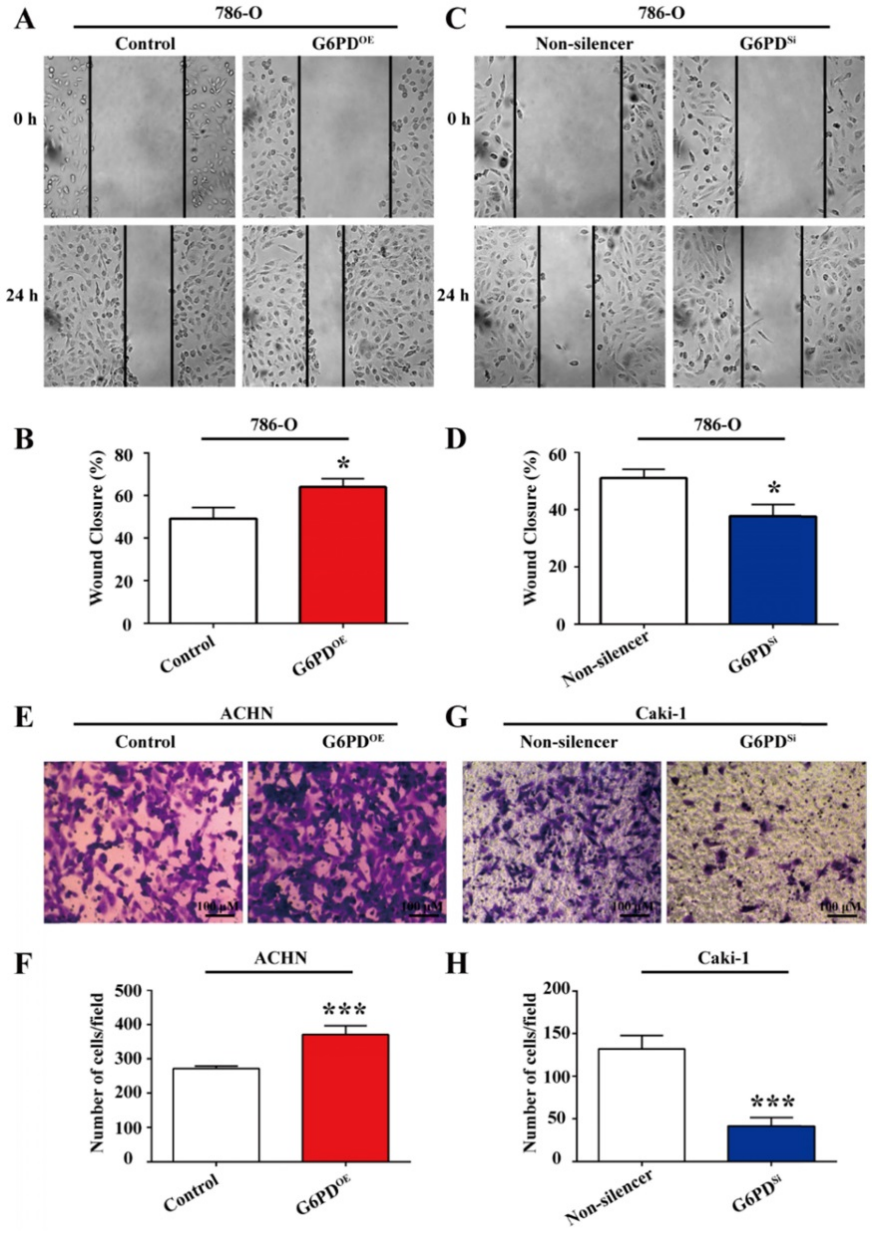
** G6PD enhances the migration ability of ccRCC cells.** (**A**) Wound-healing assay was performed to determine the effect of G6PD on migration abilities of 786-O-G6PD^OE^, 786-O- G6PD^si^ and relevant control cells. **(B)** ACHN-G6PD^OE^, Caki-1- G6PD^si^ and relevant control cells were subjected to Transwell assays. Representative images (**A, C, E, G**) and quantification analyses (**B, D, F, H**) are shown. The statistical data represented three independent experiments, each performed in triplicate. Error bars represent the means ± SD. *p <0.05, **p <0.01, ***p <0.001 vs. Control or Non-silencer group (unpaired Student's *t*-test).

Our previous study revealed that G6PD could promote ccRCC invasion through mediating MMP2 12. However, how G6PD mediates the progression of ccRCC to metastasis is still not clear. As increased cell migration was an important aspect in metastasis and positively correlated with the degree of malignancy and the mortality of ccRCC patients, wound healing assay and transwell analysis were performed using 786-O/ACHN-G6PD^OE^, 786-O/Caki-1-G6PD^Si^ and relevant control cells to evaluate whether G6PD imparted the migration ability of ccRCC cells. The results showed that wound healing ability was increased by 30.4% in 786-O-G6PD^OE^ cells at 24 hours **(Fig. 5A-B)**, while decreased about 26.2% in 786-O-G6PD^Si^ cells compared with the Non-silencer cells** (Fig. 5C-D)**. Moreover, the transwell analysis demonstrated that G6PD overexpression could increase the migration ability of ACHN-G6PD^OE^ cells by about 3.0-fold compared to that of the control **(Fig. 5E-F)**. In contrast, about 68.6% decreased cell mobility was observed in the Caki-1-G6PD^Si^ cells compared to that in the Non-silencer cells **(Fig. 5G-H)**. The above evidences indicate that G6PD could promote the migration ability of ccRCC cells.

### Cyclin E1 and MMP9 are involved in the G6PD-mediated ccRCC cells proliferation and migration

**Figure 6:**
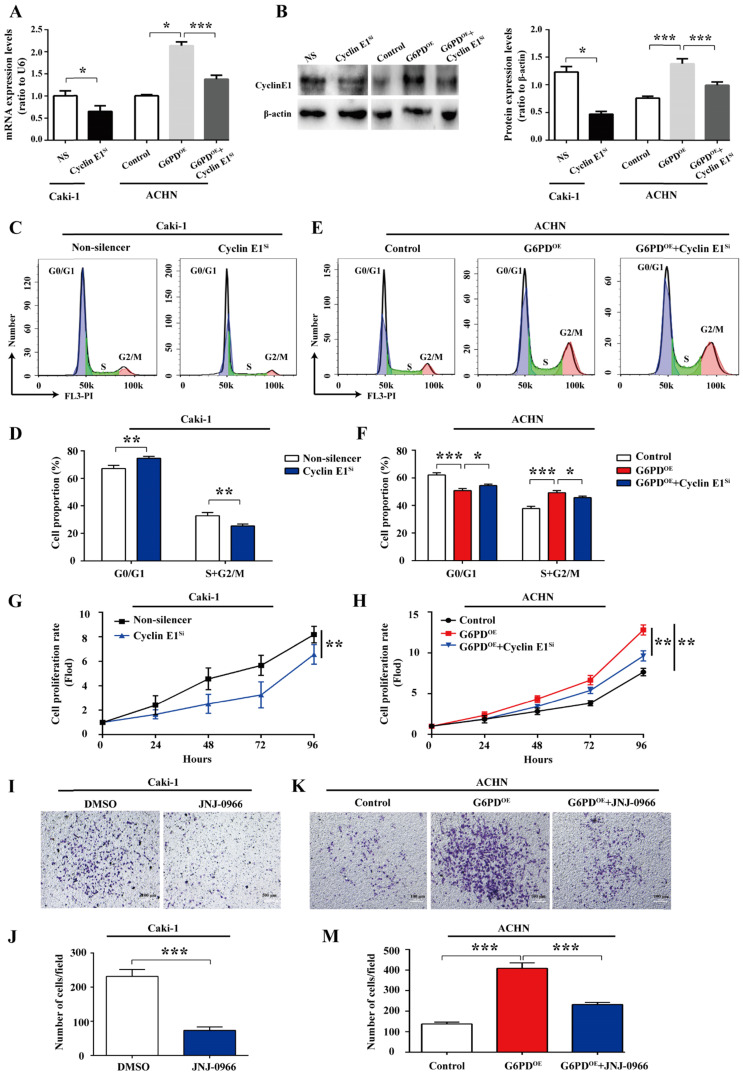
** Cyclin E1 and MMP9 are involved in the G6PD-mediated ccRCC cells proliferation and migration.** (**A-B**) The expression of Cyclin E1 at the mRNA and protein level in Caki-1, ACHN-G6PD^OE^ and relevant control cells was analyzed by using real-time RT-PCR (**A**) and Western blot assay (**B**), respectively at 48 h after Cyclin E1 siRNA transfection. β-actin was used as a protein loading control. Representative cropped gels and blots of the Western blot analysis were shown (**B**). (**C-F**) Cyclin E1 siRNA transfected Caki-1, ACHN-G6PD^OE^ and relevant control cells were subjected to cell cycle distribution analysis by PI staining and flow cytometry assay. (**G-H**) Cell proliferation abilities of Caki-1-Cyclin E1^si^, ACHN-G6PD^OE^-Cyclin E1^si^ and relevant control cell lines were assessed by MTS assay at different time points. (**I-M**) Caki-1, ACHN-G6PD^OE^ cells following treatment with the MMP9 inhibitor JNJ-0966 (10 μM, 24 h) and relevant control cells were subjected to Transwell assays. Representative images (**I**, **K**) and quantification analyses (**J**, **M**) are shown. All assays were done in at least triplicate. Bars represent the means ± SD. *p <0.05, **p <0.01, ***p <0.001 vs. relevant control (Mixed ANOVA for **G-H**, unpaired Student's *t*-test for others).

All the above results imply that G6PD-mediates proliferation and migration may be heavily dependent on the up-regulation of Cyclin E1 and MMP9 in ccRCC cells. For further clarify this notion, a series of experiments were conducted to clarify whether the Cyclin E1 and MMP9 levels could affect the G6PD overexpression facilitated ccRCC cells proliferation and migration, respectively. Caki-1 cells with relative high expression of G6PD, Cyclin E1 and MMP9 were chosen for Cyclin E1 knockdown and MMP9 activity inhibition, and subsequently for genes function detection. Whereas, ACHN-Control cells with relative low gene expression levels were used for the revers experiment along with the ACHN-G6PD^OE^ cells. Firstly, the Cyclin E1 siRNA transfection efficiency in Caki-1, ACHN-Control and ACHN-G6PD^OE^ cells was testified by real-time RT-PCR and Western blot respectively. The results demonstrated that Cyclin E1 expression levels were significantly decreased at the mRNA and protein level in both Caki-1 and ACHN-G6PD^OE^ cells (**Fig. 6A-B**). Subsequently, the cell cycle dynamics and cell proliferation rates were analyzed by using flow cytometry and MTS assay, respectively.

As presented in** Fig. 6C-D**, in Caki-1 cells, the cell population of G0/G1 phase was significantly increased by approximate 11.1% following the Cyclin E1 siRNA transfection, while the cell population of S and G2/M phases showed an obvious decrease compared to that of the control. Meanwhile, as what we confirmed previously in **Fig. 4A-B**, G6PD-overexpression (ACHN-G6PD^OE^) could result in an obvious decrease in the G0/G1 fraction and an increase in the S and G2/M phase compared to that of the control. Whereas, Cyclin E1 siRNA reversed the G6PD -overexpression promoted cell cycle progression and induced cell cycle arrest in G0/G1 phase (**Fig. 6C-D**).

The results of subsequent MTS assay showed that when Cyclin E1 was knocked down, an approximate 19.8% decreased proliferation rate was observed in Caki-1 cells at 96 hours after seeding compared to that of the Non-silencer cells **(Fig. 6G)**. Meanwhile, **Fig. 6H** confirmed that overexpression of G6PD in ACHN cells significantly increased the cell growth rate by about 0.6-fold at 96 hours after seeding compared to that of the control cells, but this cell proliferation promoting effect of G6PD-overexpression could be obviously reversed by approximate 24.7% following Cyclin E1 knockdown. The above evidences indicate that Cyclin E1 might be an important regulatory target gene of G6PD mediated signaling pathways in the proliferation of ccRCC.

Furthermore, JNJ0966, a specific therapeutic inhibitor of MMP-9, which is commonly used for many researches, including cancer, fibrosis, immune dysregulation, and neurodegenerative diseases, could inhibit activation of MMP-9 zymogen and subsequent generation of catalytically active enzyme, but have no effect on MMP-1, MMP-2, MMP-3, MMP-9, or MMP-14 catalytic activity and does not inhibit activation of the highly related MMP-2 zymogen. Therefore, JNJ0966, the highly selective compound, was used to treat ccRCC cells at the concentration of 10 μM for 24 h in our current study for MMP9 activity inhibition 22, 23. The results of subsequent transwell analysis demonstrated that about 68.3% decreased cell mobility was observed in the Caki-1 cells following treatment with the MMP9 inhibitor JNJ-0966 **(Fig. 6I-J)**, while the upregulated migration ability could be reversed by about 43.2% following JNJ-0966 stimulation in G6PD-overexpressing ACHN cells **(Fig. 6K-M)**. These results suggest that MMP9 is required for the G6PD enhanced migration ability of ccRCC cells. Taken together, these evidences suggest that Cyclin E1 siRNA and MMP9 inhibitor could reverse G6PD upregulated ccRCC cells proliferation and migration, which indicating that these functional genes including Cyclin E1 and MMP9 may probably be necessary regulatory factors and involved in the G6PD-mediated ccRCC proliferation and migration.

### G6PD upregulates Cyclin E1 and MMP9 expression in the xenografted ccRCC mice model

**Figure 7:**
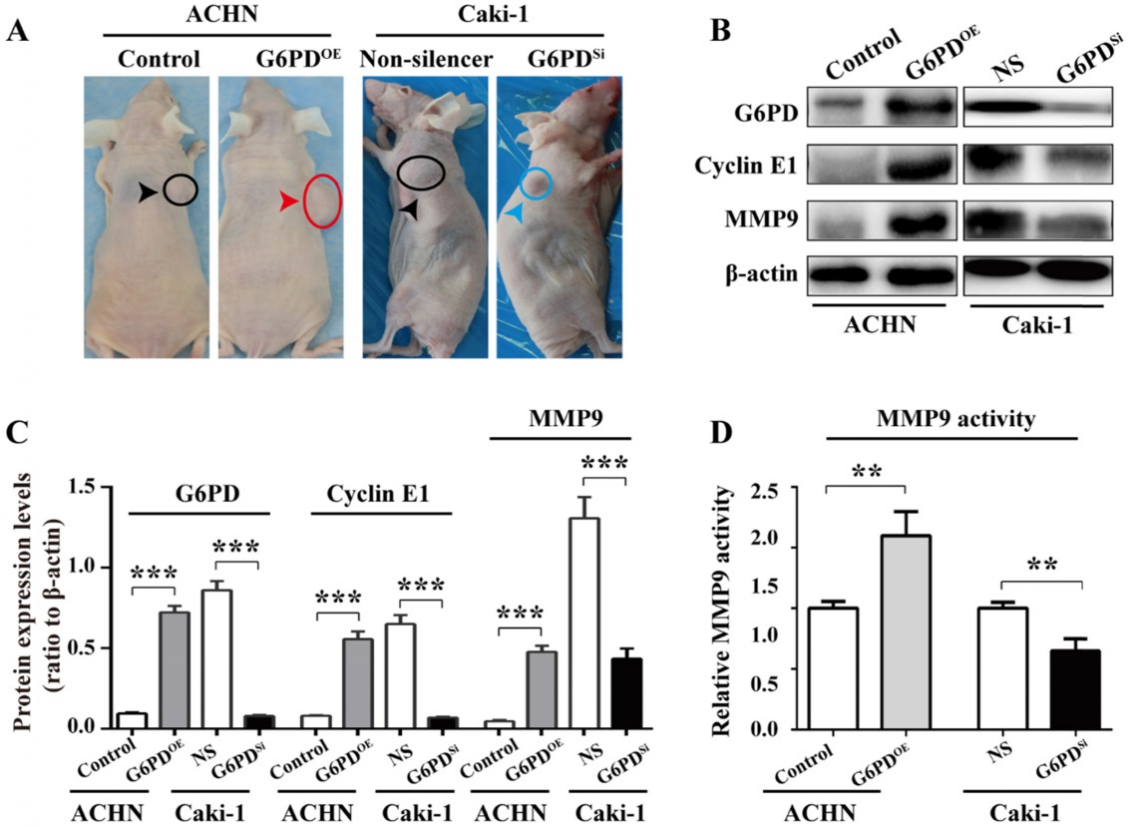
** G6PD upregulates Cyclin E1 and MMP9 to enhance ccRCC progression* in vivo*.** (**A**) Stably transfected ACHN-G6PD^OE^, Caki-1- G6PD^si^ and relevant control cells were subcutaneous injected in the nude mice, respectively. Representative xenografted mice images were shown. (**B-C**) The protein expression of G6PD, Cyclin E1 and MMP9 in the mice tumor tissue were analyzed by Western blot analysis (**B**) and grayscale scanning (**C**). β-actin served as a loading control. Representative cropped gels and blots of the Western blot analysis were shown (**B**). The samples used for quantitative comparisons in the Western blot analysis were derived from the same experiment and that gels were processed in parallel (**C**). (**D**) Relative MMP9 enzyme activities in the mice tumor tissue were analyzed by using MMP9 activity kit. The data represent three independent experiments. Each bar represented the mean ± SD. **p <0.01, ***p <0.001 vs. Control or Non-silencer (unpaired Student's *t*-test).

To further back up the importance of G6PD-mediated Cyclin E1 and MMP9 overexpression in the progression of ccRCC, *in vivo* study was conducted as described in one of our previous reports 12. Xenografted nude mice models were constructed by subcutaneously injecting ACHN-G6PD^OE^, Caki-1-G6PD^Si^ or relevant control cells. The results revealed that ACHN-G6PD^OE^ cells produced larger tumor compared with control, whereas Caki-1-G6PD^si^ parental cells induced smaller tumors compared with the Non-silencers **(Fig. 7A)**. Subsequently, the mice tumor tissues were subjected to Western blot analysis. As presented in **Fig. 7B-C**, the expression levels of G6PD, Cyclin E1 and MMP9 protein were significantly increased in ACHN-G6PD^OE^-derived tumor tissues, whereas they were obviously decreased in Caki-1-G6PD^Si^-derived tumor tissues compared with the corresponding controls. Moreover, about 0.6-fold increased MMP9 activity was detected in ACHN-G6PD^OE^-derived tumor tissues compared with the control, whereas the MMP9 activity was decreased by about 35% in Caki-1-G6PD^Si^-derived tumor tissues **(Fig. 7D)**. Taken together, these results demonstrate that G6PD upregulates Cyclin E1 and MMP9 expression in the xenografted ccRCC mice model, which indicate that G6PD may promote ccRCC progression through facilitating the expression of both Cyclin E1 and MMP9.

## Discussion

It has been reported that about 33% of RCC has already metastasized at the first diagnosis, and 20% ~ 50% of patients will progress to metastasis following surgery 1, 6. Despite ccRCC treatment has developed for decades, the advanced and metastatic ccRCCs are still challenging due to its resistance to chemo- and radiotherapy, therefore RCC patients are still confronted with worse prognosis 1. Although enormous efforts about identifying appropriate biomarkers for ccRCC tumorigenesis, progression and aggressiveness have been made to improve the efficiency of ccRCC diagnosis and prognosis, to date fewer particular biomarkers exhibit satisfactory potential for ccRCC classification and prognosis prediction or is ready for widespread use in clinical application 1, 24. Therefore, one of the main aims of present study is to investigate the underlying mechanism of ccRCC progression and identify new biomarkers that are associated with ccRCC tumor development and clinical parameters, which may be helpful for ccRCC earlier diagnosis and prognosis, and may even become novel therapeutic options and improve the survival of ccRCC patients.

The present study scrutinizes the hypothesis that the biological function and mechanism of G6PD-mediated ccRCC progression involve the modulation of Cyclin E1 and MMP9 expressions. Moreover, the results suggested that the interplay between G6PD, Cyclin E1 and MMP9 is more likely to be implicated in the development of ccRCC rather than Cyclin D1 and MMP2. G6PD was shown, in our previous study, to promote ccRCC proliferation by upregulating the Cyclin D1 expression. However, recent studies delineate controversy about the prognostic role of Cyclin D1 in RCC 25. Some study indicated that low expression of Cyclin D1 was linked to large tumor size, high nuclear grade, and poor prognosis of ccRCC patients 15. A very recent meta-analysis of 18 studies with 2282 RCC patients demonstrated that high Cyclin D1 expression level was positively associated with better prognosis of RCC patients in disease free survival rate, but there was no association between overall survival and Cyclin D1 expression in ccRCC patients 25. Our present and previous studies supported that Cyclin D1 expression is high in ccRCC and modulated by G6PD 9. However, both genes were not positively correlated, and ccRCC patients with high Cyclin D1 expression showed better prognosis. Additionally, some studies have even suggested non-oncogenic role for Cyclin D1, and down-regulated Cyclin D1 could increase the cell invasion and improve the outcome of breast cancer patients 25. These findings indicated that Cyclin D1 performed roles besides oncogenic and might exert functions in impairing the malignant potential of ccRCC.

As an important cell cycle regulator, the classic function of Cyclin D1 is to form a complex with cyclin dependent kinase (CDK) 4/6 and promote G1/S transition 25. However, the catalytic partners of Cyclin D1, CDK 4 and CDK6, did not provide satisfactory results either. We found that there was no significant difference between the expression level of CDK4 presented in ccRCC and normal tissues, whilst CDK6 expression was conversely decreased in ccRCC specimens compared with the control. Moreover, neither CDK4 nor CDK6 showed any prognostic significance on the impact of ccRCC patients' survival (data not shown). These aforementioned controversies prompt us to identify other more accurate proliferation-related factor that could be regulated by G6PD and involved in ccRCC tumorigenesis. The present study demonstrated that the G1/S transition regulator Cyclin E1 was highly expression in ccRCC and could be a potential biomarker for ccRCC prognosis. This finding is strongly consistent with previous reports that demonstrated the oncogenic function of Cyclin El in cancers. For instance, its oncogenic role was highlighted in breast cancer 26, ovarian cancer 27 and osteosarcoma 28. More interestingly, Cyclin E1 could be mediated by G6PD overexpression and high Cyclin E1 expression predicted poor outcomes, which indicated that as a cell cycle-related molecular, Cyclin E1 might be a more crucial downstream target of G6PD in promoting ccRCC tumor proliferation.

Previous study from our laboratory demonstrated that G6PD is overexpressed in ccRCC and has the ability to promote tumor cell proliferation and invasion 9, 12. However, whether G6PD could enhance ccRCC migration and the underlying regulatory mechanisms are remains unknown. MMPs are intriguing genes related to cancer progression, and they have been found to exert crucial regulatory roles in cell apoptosis, migration, angiogenesis and immunity. Increasing evidences demonstrate that MMPs are commonly upregulated in types of human cancers and associated with patient prognosis. For instance, MMP1, 3, 9 and 10-14 were highly expressed in breast cancer, colon adenocarcinoma, esophageal cancer, head and neck cancer, etc. However, some MMPs are downregulated in some cancers, such as MMP2 and 23B in breast cancer, bladder cancer, lung squamous cancer and uterine corpus endometrial carcinoma 17. The expression of representative MMPs were also measured in ACHN-G6PD^OE^, Caki-1 G6PD^Si^ and relevant control cells in our study 12. The previous and present results showed that MMP2 and MMP9 had the most significant protein expression changes when G6PD was overexpressed or knocked down in ccRCC.

MMP2 and MMP9, also known as gelatinase A and gelatinase B, are considered to be the major MMPs involved in invasion and metastasis of numbers cancers because of their capacity to degrade the important components of basement membranes, including laminin, gelatin, nidogen, type I and IV collagens 19. Nevertheless, as Cyclin D1, the prognostic role of MMP2 in RCC is controversial. Some reports showed that MMP2 is overexpressed in RCC, involved in RCC invasion and angiogenesis, and correlated with poor outcome of RCC patients 12, 29. However, our present study, together with other reports, found no significant difference of MMP2 expression in the analysis of large numbers of ccRCC clinical samples and normal kidney tissue 17. Furthermore, we found that MMP2 expression level is not associated with ccRCC prognosis** (Supplement 1)**. Otherwise, MMP9 has been found to have the largest fold change between ccRCC and normal control tissues 17. Consistent with this, our study also found that MMP9 was highly expressed in ccRCC tissues, and it predicted poor outcomes in ccRCC patients. Of note, the current study demonstrated that MMP9 expression can be regulated by G6PD in ccRCC. In fact, both genes are positively correlated in ccRCC. However, the multivariate Cox regression analysis showed that the expression MMP9 was not included in the independent prognostic factors for ccRCC survival, which suggested the complications of identifying potential biomarkers for ccRCC 24. Though the current findings do not yet provide an immediate clinical application, some essential clues are revealed and more researches are required in future investigation.

Our current study demonstrated that both MMP9 protein expression level and activity could be upregulated by G6PD through the ccRCC cell model and xenografted mice model analyses. Intriguingly, we observed that the mRNA expression of MMP9 was not obviously changed following G6PD overexpression or knockdown in ccRCC cell lines, which seemed to be inconsistent with the results of TCGA dataset mining which indicated both genes were highly expressed and positively correlated at the mRNA level in ccRCC tumor specimens and normal control.

The possible reasons for these inconsistences were as follows. Firstly, the TGGA dataset was dependent on the transcriptome sequencing, whereas, our mRNA detection in G6PD overexpressing or knocked down ccRCC cells was conducted by real-time RT-PCR analysis. Both of these two different methods and currently available other technologies are perfectly accurate in neither mRNA or protein quantifications. These factors potentially influence the detection of biologically truly significant correlations 30. Secondly, the TCGA transcriptional expression analyses of G6PD and MMP9 were test results of human ccRCC tumor specimens and normal control kidney tissues, which were different from the detection of mRNA and protein expression levels based on stably transfected cell models. Therefore, different biological or experimental mRNA and protein degradation rates might affect the mRNA and protein correlations 30. Thirdly, gene expression could be regulated at several levels including pre-transcriptional, transcriptional and post-transcriptional levels, and any variation in regulatory link, zymogen activatiocn, enzyme concentration and hence activity may result in enhanced MMP9 function of promoting tumor migration and progression in ccRCC. Although some gene mRNA was detected, there may be no indication that the mRNA was translated into protein 31, and vice versa, in some cases, high expression levels of protein may lead to stress response which results in decreased mRNA transcription 31, 32. Therefore, some mRNA samples allow good estimations about the corresponding protein expression, but for some others, pronounced deviation could be observed 33, 34.

Protein and functional activity of a gene showed a significant correlation in most cases, but there could be no correlation between any of these parameters and mRNA levels, such as the cytochrome P4502E1, a constitutively expressed gene in human liver 32, 34. In our present study, G6PD upregulated protein expression and functional activity of MMP9 demonstrated that G6PD could facilitate ccRCC progression partially through promoting MMP9 function. However, the inconsistent expression of MMP9 gene at the mRNA and protein levels, when G6PD was overexpressed or knocked down in ccRCC cell lines, might be depended on the stress caused by exogenous gene transfection. When level of one protein was too high, it may cause some kind of stress on cell. To maintain balance within cells and save energy, cells inevitably reduced the gene transcription, whereas, cells may promote transcription, when low level of protein was translated 30, 32, 34. Compared with the different testing object from TCGA transcriptome sequencing, stable transfected ccRCC cell lines were stressed by G6PD OE/KD and the following significant modification of MMP9 protein and activity, which may rather result in little change of MMP9 at the mRNA levels.

Taken together, the results of TCGA dataset mining indicated that G6PD, Cyclin E1 and MMP9 were overexpressed and positively correlated in ccRCC, which were just hints, but not causation. The present study scrutinizes the hypothesis that the biological function and mechanism of G6PD-mediated ccRCC progression involve the modulation of Cyclin E1 and MMP9 expressions. The little changes of MMP9 at the mRNA level in ccRCC cell models does not affect our core conclusion of this report. However, it is an interesting phenomenon and meaningful work that needs further study.

Thus, our present study, supported with strong methodology, provide novel therapeutic pathway, involving G6PD, Cyclin E1 and MMP9, that can be considered in future for ccRCC treatment. It is well established that G6PD is critical in the maintenance of the redox equilibrium in the cell. It preserves the cell homeostasis by regulating ROS production and elimination 11. Doing so, G6PD sustains the high level of ROS in cancer cells while instigating their survival. In fact, it has been reported that ROS dysregulation is an important factor leading to abnormal signal transduction in cells 35, 36. ROS promotes and interacts with numerous oncogenic signaling pathways, such as the STAT3, MAPK and NF-κB pathways, to favor the development of human cancers. Moreover, proliferation and metastasis related genes, such as cyclins and MMPs, which were transcriptional regulation targets of these oncogenic signaling transducers 37-40 and were found to be involved in this ROS-mediated mechanisms of action in ccRCC 12, 21, 41. These implies that ROS and its relevant signaling pathways might be involved in the G6PD-mediated upregulation of Cyclin E1 and MMP9 in our study. However, to elucidate the exact regulatory mechanism, some more *in vitro* and *in vivo* experiments should be conducted in the future investigation.

Intriguingly, we observed that the mRNA expression of MMP9 was not significantly modified when G6PD was overexpressed or knocked down. We hypothesized that it may be resulting from some epigenetic modifications. The absence of mRNA-protein correlation for a subset of investigated genes suggested that the relation between mRNA and protein was not strictly linear, but had a more intrinsic and complex dependence, deviating from the classical view referred to as the molecular dogma. Different regulation mechanisms, such as synthesis and degradation rates, acting on both the synthesized mRNA and the synthesized protein, affected the amount of the two molecules differentially 30, 32, 34. In the current study, G6PD probably increased MMP9 protein stability through some intermediate mediator and other regulatory mechanisms. Additionally, it has been proved that ROS can activate MMPs or regulate MMP9 mRNA stability and lead to the destruction of extracellular matrix and facilitate tumor metastasis 19, 42, which promote us to hypothesize that facilitated MMP9 activation in ccRCC may also be induced by the G6PD-mediated ROS accumulation. Moreover, it has been well confirmed that G6PD was involved in certain carcinogenesis, and served key roles in extensive cancer cell metabolic reprogramming, including affection the amino acid metabolic pool 43-45, which may in turn provide building blocks for MMP9 protein synthesis. Hence, how G6PD, a cytoplasmic enzyme, regulates Cyclin E1 and MMP9 overexpression, and which signaling pathway serves as a mediator between these aberrations are unknown and required to be clarified in future studies.

## Conclusion

In summary, the present study corroborates the oncogenic role of G6PD in ccRCC and extends the involved molecular mechanisms. The results indicated that G6PD changed cell cycle dynamics, facilitated cell proliferation, promoted migration *in vitro*, and enhanced ccRCC tumor growth *in vivo*, probably by upregulating Cyclin E1 and MMP9. Moreover, G6PD was positively correlated with Cyclin E1 and MMP9, all being highly expressed in human ccRCC tissues and associated with poor ccRCC prognosis. These findings reveal the feasibility of G6PD, Cyclin E1 and MMP9 as novel biomarkers and pave ways for the development of novel therapeutics for ccRCC.

## Supplementary Material

Supplementary figure.

## Figures and Tables

**Table 1:** Correlations between the expression of Cyclin D1, Cyclin E1, MMP9 and important clinicopathological variables in ccRCC.

Parameters	Case No.	Cyclin D1		Cyclin E1		MMP9	
		Expression	*P* value	Expression	*P* value	Expression	*P* value
**Sex**							
Male	344	7.040	**<0.001^a^**	0.732	0.109^a^	2.559	0.227^a^
Female	184	7.428		0.710		2.436	
**Age**							
< 60	245	7.124	0.194^a^	0.717	0.439^a^	2.417	0.316^a^
≥ 60	283	7.219		0.732		2.601	
**T stage**							
T1/2	340	7.326	**<0.001^a^**	0.663	**<0.001^a^**	2.215	**<0.001^a^**
T3/4	188	6.902		0.836		3.060	
**N stage**							
N0	239	7.145	**0.003^b^**	0.701	**<0.001^b^**	2.597	0.096^b^
N1	16	6.364		1.257		3.210	
Nx	273	7.248		0.714		2.405	
**M stage**							
M0	420	7.269	0.064^b^	0.678	**<0.001^b^**	2.498	**<0.001^b^**
M1	78	6.681		0.857		2.930	
Mx	28	7.174		0.768		1.539	
NA.	2	6.602		0.655		3.783	
**Laterality**							
Right	279	7.244	0.583^b^	0.694	0.050^b^	2.493	0.469^b^
Left	248	7.097		0.759		2.547	
Bilateral	1	7.193		0.912		1.140	
**Fuhrman grade**							
G1/2	240	7.433	**<0.001^b^**	0.617	**<0.001^b^**	2.177	**<0.001^b^**
G3/4	280	6.984		0.801		2.824	
Gx	5	5.661		0.839		1.634	
NA.	3	6.909		2.060		2.326	
**TNM stage**							
I/II	320	7.358	**<0.001^b^**	0.658	**<0.001^b^**	2.200	**<0.001^b^**
III/IV	205	6.895		0.827		2.992	
Discrepancy	3	6.778		0.895		3.663	
**G6PD expression**							
Low	264	6.341	**<0.001^a^**	0.458	**<0.001^a^**	1.253	**<0.001^a^**
High	264	8.009		0.991		3.779	

Abbreviations: NA., Not Available. ^a^, Mann-Whitney U test; ^b^, Kruskal-Wallis H (K) test. Significant *p*-value was in **bold**.

**Table 2:** Univariate and multivariate Cox regression analyses of the association of G6PD, Cyclin D1, Cyclin E1 and MMP9 expression and other clinicopathologic features with overall survival in ccRCC.

Characteristics	Univariate			Multivariate		
	HR	95% CI	*p* value	HR	95% CI	*p* value
**Sex**	0.952	0.698-1.297	0.753			
**Age**	1.786	1.304-2.447	**<0.001**	1.638	1.190-2.255	**0.002**
**T stage**	3.021	2.232-4.089	**<0.001**	0.704	0.402-1.236	0.222
**N stage**	0.914	0.786-1.063	0.243			
**M stage**	2.131	1.693-2.681	**<0.001**	1.493	1.135-1.965	**0.004**
**Laterality**	1.393	1.034-1.877	**0.029**	1.339	0.993-1.806	0.056
**Fuhrman grade**	2.142	1.602-2.864	**<0.001**	1.379	0.980-1.942	0.065
**TNM stage**	3.699	2.730-5.012	**<0.001**	2.583	1.853-3.601	**<0.001**
**G6PD expression**	1.959	1.547-2.482	**<0.001**	1.423	1.070-1.892	**0.015**
**Cyclin D1 expression**	0.770	0.682-0.871	**<0.001**	0.975	0.834-1.140	0.751
**Cyclin E1 expression**	1.713	1.422-2.062	**<0.001**	1.447	1.113-1.881	**0.006**
**MMP9 expression**	1.193	1.099-1.295	**<0.001**	1.030	0.935-1.135	0.546

Abbreviations: HR, hazard ratio; CI, confidence interval. Significant *p*-value was in **bold**.
