# Supplementary material for: G6PD upregulates Cyclin E1 and MMP9 to promote clear cell renal cell carcinoma progression
Source: Int J Med Sci. 2022 Jan 1;19(1):47–64. doi: 10.7150/ijms.58902 (PMC8692124; doi:10.7150/ijms.58902)
Supplement: Supplementary file 1 — Supplementary figure. [file ijmsv19p0047s1.pdf]

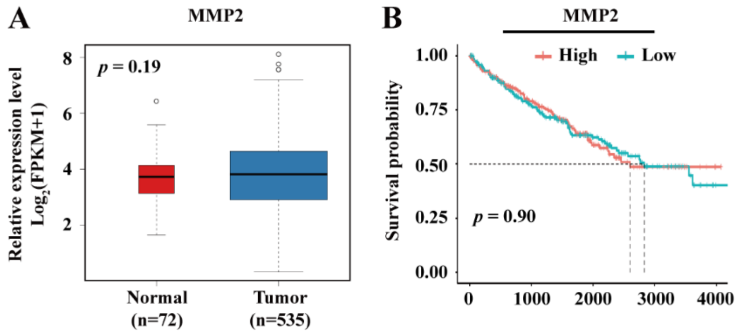

Supplement 1. The expression prolife and prognostic significance of MMP2 in ccRCC. (**A**) mRNA expression levels of MMP2 in normal kidney tissues (n=72) and ccRCC specimens (n=535) were analyzed by TCGA dataset mining (Mann-Whitney U test). (**B**) Kaplan-Meier analysis was conducted for overall survival of ccRCC patients in the TCGA cohort with high vs. low MMP2 mRNA expression levels (log-rank test).
